# Challenges to implementing mandatory hepatitis B vaccination: bridging immunization gaps among health workers in sub-Saharan Africa

**DOI:** 10.1186/s41182-025-00712-w

**Published:** 2025-03-05

**Authors:** Stephen Olaide Aremu, Akyala Ishaku Adamu, Babatunde Fatoke, Legbel Ikenna Uguru, Samuel Olusegun Itodo, Dorcas Oluwakemi Aremu, Deborah Bukola Aremu, Abdillahi Abdi Barkhadle

**Affiliations:** 1 Global Health and Infectious Control Disease Institute, Nasarawa State University, Keffi, Nasarawa State Nigeria; 2 General Hospital Lagos, Lagos Island, Odan, Lagos State Nigeria; 3 Benue State University, Makurdi, Benue State Nigeria; 4 Sechenov University, Moscow, Russian Federation; 5 Federal University of Technology, Minna, Nigeria; 6 Rift Valley University, Addis Ababa, Ethiopia

**Keywords:** Hepatitis B virus (HBV), Hepatitis B vaccine (HepB), Healthcare workers (HCWs), Vaccination gaps, Mandatory vaccination, Sub-Saharan Africa (SSA), Immunization

## Abstract

Hepatitis B virus (HBV) poses a major occupational risk for healthcare workers (HCWs) in sub-Saharan Africa (SSA), where endemicity and resource limitations hinder effective prevention. Mandatory Hepatitis B vaccination (HepB) policies for HCWs are critical to addressing vaccination gaps, yet their implementation is challenged by dosing inconsistencies, lack of post-vaccination immunity testing, and ethical considerations. In many countries in SSA, adults are given a four-dose pediatric formulation of the vaccine due to limited access to adult formulations that require fewer doses, creating logistical challenges and increasing the risk of incomplete immunization. In addition, the absence of routine post-vaccination immunity testing undermines efforts to ensure adequate protection, leaving vaccinated HCWs potentially vulnerable to HBV infection. Ethical issues, including inadequate informed consent, out-of-pocket vaccination costs, and inequities in access, further complicate mandatory vaccination programs. Addressing these barriers requires a multi-sectoral approach, including increased access to adult formulations of vaccines, integration of immunity testing into vaccination protocols, subsidized vaccine costs, and culturally tailored education campaigns. These strategies are essential for ensuring that vaccination policies are effective, equitable, and ethical. Protecting HCWs against HBV not only safeguards their health but also strengthens healthcare systems and public health outcomes in SSA.

Hepatitis B virus (HBV) poses a major public health challenge globally, with sub-Saharan Africa (SSA) being among the regions most affected [1, 2]. Vaccination coverage in SSA remains inequitable, particularly for HBV. Two-thirds of new cases originate in SSA according to the Global Hepatitis Report 2024 [2]. In children, coverage for the Hepatitis B Vaccine (HepB) birth dose varies widely across SSA, ranging from less than 10% in some countries to over 80% in others, while adults typically have lower vaccination rates due to limited adult immunization programs and awareness campaigns [3–7]​. Among healthcare workers (HCWs), the coverage is often suboptimal due to limited access to adult vaccine formulations, vaccine sites for those in rural areas, limited availability, vaccine hesitancy, and systemic challenges [3–7]. For example, some studies have reported HepB vaccination prevalence as low as 18% in healthcare workers in SSA, with significant gaps in receiving the full vaccine series [7]. HCWs in SSA face a heightened risk of occupational exposure due to the high prevalence of HBV in the population and limited access to protective resources such as personal protective equipment [8–10]. Vaccination is a cornerstone of HBV prevention, yet many HCWs in the region remain unvaccinated or only partially vaccinated. While mandatory vaccination policies for HCWs may appear to be a practical solution, mandating vaccine implementation is fraught with challenges, including vaccine dosing inconsistencies, lack of post-vaccination immunity testing, and ethical concerns [10]. Addressing these issues is crucial to achieving the dual goals of protecting HCWs and strengthening public health systems in SSA [11].

One of the most pressing issues in HBV vaccination efforts for HCWs in SSA is the use of pediatric dose formulations instead of adult dose formulations. In many countries across the region, healthcare systems are constrained by limited access to vaccine formulations tailored for adults. As a result, pediatric formulations are repurposed for HCWs. However, these pediatric formulations named Engerix-B or recombivax (0.5 mL) often require four injections (including booster dose) spread across an extended schedule (a 0-, 1-, 6-, and 12-months), in contrast to the standard two-dose formulations (0 and 1 month) adult regimen named Heplisav (1 mL) given intramuscularly, which is not available in SSA and is only for adults [12]. This dosing inconsistency creates logistical challenges, as the extended schedule requires multiple follow-ups, which are difficult to achieve in resource-constrained settings. In practice, HCWs may fail to complete the full pediatric vaccination course, which may leave them inadequately protected [13]. The reliance on pediatric doses also highlights broader systemic issues, such as the lack of adequate planning and procurement to meet adult vaccine demands. Without targeted investments to address this gap, vaccination programs risk perpetuating inefficiencies. To ensure effective immunization, governments must prioritize the procurement of adult formulations of HepB vaccines, which not only provide better protection for HCWs but also simplify follow-up by reducing the number of required doses [14]. Bulk purchasing agreements, partnerships with global health organizations, and streamlined supply chains could play pivotal roles in resolving this challenge [15].

Another critical challenge undermining the effectiveness of HBV vaccination programs in SSA is the lack of routine post-vaccination immunity testing. Anti-HBs (hepatitis B surface antibody) testing is essential to confirm that individuals have developed adequate immunity following vaccination [16], considering that a certain percentage of people are baseline non-responders, and literature does not exist to describe the percentage of “non-responders” in LMICs which is estimated to be between 5 and 30% in studies of the United States and Scandinavia, many of whom do not respond even after re-dosing. This is a complicated issue that could be difficult to address in low- and middle-income countries (LMIC) [17]. Unfortunately, most healthcare systems in SSA lack the infrastructure and resources to conduct these tests. This omission creates a significant gap, as vaccinated HCWs may remain unaware that they are adequately protected, leaving them vulnerable to HBV infection despite their compliance with vaccination protocols. The absence of immunity testing underscores the need for more comprehensive vaccination policies that incorporate post-vaccination monitoring as a standard practice. Investments in laboratory infrastructure and training for healthcare personnel are necessary to make post-vaccination testing accessible and affordable. Although the feasibility of anti-HBs testing is challenging in many resource-limited settings, donor organizations, governments, and global health institutions must collaborate to fund and implement these measures, ensuring that HCWs receive not only the vaccine but also the assurance of its efficacy, alternatively, conducting research on immunity outcomes and the efficacy of different dosing regimens may offer a more cost-effective, scalable, and practical solution in SSA.

Beyond logistical and infrastructural barriers, mandatory HepB vaccination policies in SSA are also fraught with ethical challenges [18]. One of the primary ethical concerns is the issue of informed consent. In many cases, HCWs are required to comply with vaccination mandates without adequate information about the vaccine’s benefits, potential side effects, and the importance of completing the full schedule. This lack of awareness can create a sense of coercion, undermining the principles of autonomy and voluntary decision-making [19]. Effective vaccination policies must therefore be accompanied by robust communication and educational campaigns that provide HCWs with the knowledge they need to make informed choices. Another ethical dilemma arises from the cost of vaccination. In many SSA countries, HCWs are expected to pay for their HepB vaccine formulations out-of-pocket. This practice disproportionately affects low-income HCWs, many of whom work in underfunded healthcare facilities with minimal support, alongside those with disproportionate access within low-and middle-income countries in SSA where those without the financial means cannot be protected [20]. Requiring HCWs to bear the financial burden of vaccination not only creates barriers to access but also raises questions about the fairness of mandatory policies. Governments and healthcare institutions must address this issue by subsidizing or fully covering the cost of HepB vaccination for HCWs, thereby ensuring equity and compliance.

Furthermore, there is the issue of neglect of certain categories of HCWs particularly informal and low-cadre workers, such as cleaners and porters, who often face occupational exposure yet remain excluded from vaccination programs. Similarly, private-sector HCWs and temporary staff may lack access due to inconsistent enforcement. Rural healthcare workers, students in clinical training [21], and migrant or volunteer HCWs also experience disparities in vaccine coverage. Addressing these gaps requires inclusive policies and equitable resource allocation to ensure that all HCWs, regardless of status or location, receive adequate protection against HBV.

Mandatory HepB vaccination policies also raise concerns about the broader systemic barriers that HCWs face. It is ethically problematic to impose vaccination requirements without simultaneously addressing the underlying issues of vaccine availability, dosing inconsistencies, and immunity testing. Policies that focus solely on compliance without tackling these systemic challenges risk exacerbating existing inequalities within the healthcare system. A more holistic approach is needed, one that combines mandatory vaccination with structural reforms to ensure that HCWs receive the full benefits of immunization. Despite these challenges, the potential benefits of closing vaccination gaps among HCWs in SSA cannot be overstated. HCWs are not only at the frontline of patient care but also play a critical role in preventing the spread of infectious diseases within healthcare settings and the broader community. Ensuring that they are protected against HBV is therefore essential for safeguarding both individual and public health.

Addressing the challenges of mandatory HBV vaccination requires a multi-sectoral approach. Governments must take the lead in prioritizing HepB vaccination for HCWs, supported by technical and financial assistance from international organizations. At the same time, healthcare institutions must invest in education and training to ensure that HCWs understand the importance of vaccination and are equipped to complete the required protocols. Lastly, ethical oversight must be integrated into vaccination policies to ensure that they are fair, transparent, and responsive to the needs of HCWs.

In conclusion, mandatory HepB vaccination for HCWs in sub-Saharan Africa is an essential step in addressing occupational risks and reducing HBV transmission. However, the success of such policies depends on resolving critical challenges related to dosing inconsistencies, post-vaccination immunity testing, and ethical considerations. By investing in vaccine accessibility, infrastructure, and education, and by addressing the systemic barriers that undermine vaccination efforts, SSA can make significant strides in protecting its healthcare workforce and strengthening public health systems. Protecting HCWs is not just a matter of individual safety; it is a cornerstone of building resilient healthcare systems capable of addressing the region’s pressing health challenges.

## Data Availability

Data sharing is not applicable to this article as no datasets were generated or analyzed during the current study.

## Abbreviations

HBV: Hepatitis B virus
HepB: Hepatitis B vaccines
HCWs: Healthcare workers
SSA: Sub-Saharan Africa
LMICs: Low- and middle-income countries
Anti-HBs: (Hepatitis B surface antibody)
